# Hyperbaric oxygen therapy for chronic post-concussive syndrome

**DOI:** 10.1186/2045-9912-4-8

**Published:** 2014-04-09

**Authors:** Matthew C Davis, Mohammadali M Shoja, Shane R Tubbs, Christoph J Griessenauer

**Affiliations:** 1 Department of Neurological Surgery, University of Alabama at Birmingham, Birmingham, AL, USA; 2 Department of Neurosurgery, Children’s of Alabama, Birmingham, AL, USA

**Keywords:** Hyperbaric oxygen, Traumatic brain injury, Outcome

## Abstract

In this editorial, the value of hyperbaric oxygen therapy in the management of chronic post-concussive syndrome following mild traumatic brain injury is discussed.

## 

Traumatic brain injury (TBI) affects roughly 10 million people worldwide each year, and is a leading cause of death and disability among all demographics, disproportionately affecting young adults [1]. TBI is defined as an external mechanical insult to the brain in the form of penetration, acceleration, rotational forces, or blast waves resulting in temporary or permanent impairment in brain function. TBI is further categorized by etiology, damaged area of the brain, and severity (mild, moderate or severe). In the United States, 80% of TBI cases are mild, with loss of consciousness less than thirty minutes, retrograde amnesia of less than one day and Glasgow Coma Scale (GCS) on presentation of 13–15 [2].

Following even mild TBI, many patients experience a range of symptoms grouped together under the heading of post-concussive syndrome (PCS). Symptoms include alterations in executive function, depression, anxiety, headache, dizziness, and impairment in memory. Approximately one in five patients with PCS will experience these symptoms for greater than six months following their injury [3]. PCS is a clinical diagnosis, and many patients with mild TBI will have normal-appearing conventional CT and MRI scans. Specialized functional or metabolic sequences such as SPECT (Single Photon Emission Computed Tomography), functional MRI, or CT perfusion may detect subtle impairments in regional activity to explain the post-concussive phenomenon [4].

Therapy for post-concussive syndrome is typically supportive. Physical, occupational and behavioral therapies are the mainstay of treatment, and the vast majority of patients return to their neurologic and psychological baseline in under a year. Recently, hyperbaric oxygen therapy (HBOT) has been used as a potential therapeutic option for patients experiencing chronic PCS symptoms [5]. HBOT involves the administration of oxygen at supra-atmospheric pressures and increased fractions of inspired air.

HBOT is increasingly used to treat the long-term sequelae of a variety of neurologic conditions, including ischemic stroke, subarachnoid hemorrhage, and recently mild TBI [5-7]. HBOT is thought to induce neuroplasticity, support repair of injured brain tissue, and stimulate angiogenesis [8]. Although considered a largely benign intervention, increasing pressures beyond four to five absolute atmospheres is associated with oxygen toxicity and lowering of the seizure threshold [9]. Due to its favorable safety profile, limitations to the use of HBOT are mainly financial and practical. Therapeutic sessions require a significant time investment on the part of the patient, and a single session typically costs several hundred dollars [10].

Treatment pdigms are not standardized across studies on the use of HBOT in TBI. Atmospheric pressure, length of treatment, and number of sessions all vary between studies. With rare exception, 100% oxygen is used [11]. A typical HBOT session might consist of thirty to sixty minutes administration of 100% oxygen at 1.5 to 2.5 times atmospheric pressure. Patients typically undergo twenty to sixty sessions over four to twelve weeks, based on the condition being treated [12]. The optimal pdigm for HBOT in TBI remains to be determined.

Invasive and expensive interventions such as HBOT are associated with a greater placebo effect than benign and cheap interventions [13]. Additionally, blinded studies on HBOT are difficult to perform, as patients are able to sense even minor changes in atmospheric pressure. In a recent study, Boussi-Gross et al. attempt to address this issue through the use of a prospective, randomized, crossover trial to evaluate the impact of HBOT on patients with chronic post-concussive syndrome years after mild TBI [14]. Fifty six patients with PCS of greater than one year’s duration were randomized to receive forty HBOT sessions either before or after a two month control period. Patients were evaluated by standardized cognitive testing, quality-of-life questionnaires, and SPECT imaging at several points during the study period. Both groups showed statistically significant improvements in cognitive function, quality of life, and fronto-temporal perfusion on SPECT imaging following HBOT, but not following the control period. All SPECT analysis was blinded to the laboratory and clinical data, and showed increased brain perfusion in regions critical for higher-order cognition and memory.

While the study design allowed for both inter- and intra-group comparisons before and after HBOT administration, it did not completely address the potential role of the placebo effect in these patients. Other investigators have utilized control groups in which patients were randomized to increased atmospheric pressures with a normal atmospheric partial pressure of oxygen. In general, these studies have not shown a benefit of either increased oxygen tension or increased pressures to 1.5 to 2 times atmospheric pressure [15,16]. However these studies do not address any potential therapeutic benefit of higher pressures in the absence of increased oxygen tension. As of 2012, the Cochrane Collaboration stated that HBOT could not be routinely recommended for PCS following TBI [17]. Inadequate power in many studies, variable therapeutic protocol, and methodological flaws all prevented a positive recommendation.

Nevertheless, the study of Boussi-Gross et al. provides a compelling argument that whatever the underlying therapeutic mechanism, patients show improved cognitive function and cerebral perfusion after HBOT, and report improved quality of life. PCS is a debilitating complication of mild TBI, currently without any definitive treatment options. These results are worth future investigation in the form of larger randomized, blinded controlled trials, as well as longer follow-up periods to determine the durability of any HBOT effect.

## Competing interest

The authors declare that they have no conflict of interest in connection with this manuscript.
